# Accuracy of Multi-echo Dixon Sequence in Quantification of Hepatic Steatosis

**DOI:** 10.7759/cureus.7103

**Published:** 2020-02-25

**Authors:** Isil Yurdaisik, Fuad Nurili

**Affiliations:** 1 Radiology, Istinye University Gaziosmanpasa Medical Park Hospital, Istanbul, TUR; 2 Radiology, Memorial Sloan Kettering Cancer Center, New York, USA

**Keywords:** non-alcoholic fatty liver disease, magnetic resonance spectroscopy, proton density fat fraction, elastography

## Abstract

Objective

Today, a biopsy is the gold standard in the diagnosis of non-alcoholic fatty liver. However, a biopsy is an invasive technique, limited to the sample taken, and it may lead to misdiagnosis. Therefore, novel noninvasive options are needed. The objective of this study was to investigate the accuracy of magnetic resonance (MR) Dixon sequence and elastography using magnetic resonance spectroscopy (MRS) as a reference in the quantification of hepatic steatosis.

Methods

A total of 60 patients were included in the study. All patients underwent magnetic resonance imaging (MRI), MRS, and elastography in order to quantify hepatosteatosis. MRI and MRS imaging studies were performed using MR Dixon and high-speed T2-corrected multiple-echo 1H-MRS sequence (HISTO) sequences, respectively, in order to calculate proton density fat fraction (PDFF) values.

Results

The mean MRI-PDFF value with the MRS region of interest (ROI) was found as 9.4% ± 12.1%. The mean MRS-PDFF was found as 8.9% ± 11.3%. No statistically significant difference was found between MRS-PDFF and MRI-PDFF values measured in ROI (p < 0.005). The correlation between MRS-PDFF and MRI-PDFF was examined with Spearman’s correlation analysis. Accordingly, there was an excellent correlation between MRS and MRI values measured in ROI (r ≥ 0.8, p < 0.001). Sensitivity, specificity, positive predictive value, and negative predictive value were calculated as 96%, 100%, 89.5%, and 92.6%, respectively, for MRI-PDFF in predicting hepatic steatosis for the same ROI localization with MRS. The optimum cut-off value of MRS-PDFF in predicting hepatic steatosis was found as 5.3% using the same ROI localization with MRS.

Conclusion

The results of this study indicated an excellent correlation between MRI-PDFF and MRS-PDFF. The multi-echo Dixon MRI technique seems a promising alternative method in the detection of non-alcoholic fatty liver disease.

## Introduction

Non-alcoholic hepatic steatosis is a disease characterized by the accumulation of triacylglycerol (TAG)-rich macrovesicular or microvesicular lipid droplets in hepatocytes [1]. Non-alcoholic fatty liver disease (NAFLD) is defined as at least 5% of hepatocytes containing intrahepatic TAG or lipid vacuoles in the absence of alcohol-abusing or viral infections. Hepatic steatosis is classified as Grade 0 (healthy, < 5%), Grade I (mild, 5% - 33%), Grade II (moderate, 34% - 66%), and Grade III (severe, < 66%), depending on the fat percentage in the hepatocytes [2]. Steatosis may progress to nonalcoholic steatohepatitis, cirrhosis, or hepatocellular carcinoma. Hepatic steatosis is also a risk factor for liver transplantation and chronic kidney disease [3]. The clinical importance of fatty liver disease has resulted from its high prevalence in the general population, a wide spectrum of risk factors, and its potential of progression to cirrhosis or hepatocarcinoma. Because of the use of highly variable and subjective diagnostic criteria, the prevalence of hepatic steatosis has been reported between 3% and 39% [4-5].

The most reliable method for the detection of fatty liver is hepatic needle biopsy [6]. Today, a biopsy is the gold standard in the diagnosis of fatty liver disease. However, a biopsy is an invasive technique, limited to the sample taken, and it may lead to misdiagnosis [7]. Biopsy outcomes can differ depending on the area where the sample is taken. In addition, the volume of the typical liver piece analyzed is about 1/50,000 of the total volume of the organ [8]. Therefore, less invasive methods are needed. Ultrasonography (US) is currently the basic imaging system used for the detection of hepatic steatosis. US is an inexpensive and easily available method without radiation exposure. However, US cannot quantify liver fat and it has low sensitivity and specificity in predicting mild steatosis. Computed tomography (CT) also can rapidly detect hepatic steatosis. However, it requires radiation exposure and its diagnostic accuracy is low in predicting mild steatosis.

Magnetic resonance spectroscopy (MRS) has been used since 2005 in order to determine hepatic triglyceride concentration through the difference between resonance frequencies of water and fat [9]. Today, MRS is thought of as the non-invasive gold standard in the quantification of hepatic steatosis. However, MRS is limited to spatial coverage and is difficult to perform and analyze. Proton density fat fraction (PDFF) is shown as a practical alternative to MRS as a promising technique, which covers entire liver volume in a single breath-hold. Some studies have investigated the multi-echo Dixon method using MRS as the reference in order to quantify the fat content of the liver [10]. These studies have demonstrated that both methods eliminated the need for hepatic biopsy when PDFF was < 5%. In recent years, elastography has been successfully used to determine tissue stiffness in various organs, such as breast, tendons, and liver [11]. Preliminary reports have shown that changes in the liver parenchyma can be detected using the shear wave elastography technique. Elastography quantifies liver fat by measuring the propagation speed of ultrasound waves crossing the liver. As fibrosis progresses, liver tissue becomes stiff and the waves propagate more rapidly. It is possible to determine the degree of stiffness, and thus, the stage of the fatty liver, based on the propagation speed of the waves.

The objective of this study was to investigate the accuracy of magnetic resonance Dixon (MR Dixon) sequence and elastography using MRS as a reference in the quantification of the hepatic steatosis form of non-alcoholic fatty liver disease.

## Materials and methods

A total of 60 patients who were referred to our clinic for liver imaging investigations between December 2019 and February 2020 accepted to participate and were included in this study. The patients’ demographic data, such as age and gender, body mass index, and biochemical analysis outcomes were recorded. In addition, grades of hepatic steatosis were determined during the first US examinations and recorded. All patients underwent magnetic resonance imaging (MRI), MRS and elastography in order to quantify hepatosteatosis.

Patients with type I diabetes, drug-induced hepatitis, chronic liver disease, hepatic virus infection, a history of alcohol abuse with higher than 210 g a week in men and 140 g a week in women, and those with MRI contraindications, such as metallic implants and claustrophobia were excluded from the study. MRI and MRS imaging studies were performed using MR Dixon and high-speed T2-corrected multiple-echo 1H-MRS (HISTO) sequences, respectively, in order to calculate PDFF values.

MRI

Multi-echo gradient-echo sequences were performed with T2 correction [12]. In order to reduce the effects of T1 weighting, a low flip angle (4°C) was used [13]. Six fractional echo magnitude images were acquired during breath-holding. The center of the liver, coil, and magnetic area were aligned before the screening. A screening Dixon sequence was used to roughly and rapidly measure hepatic fat fraction. Water images, fat images, MRI-PDFF maps, and MR Dixon screening reports were obtained automatically. Screening findings were determined as normal or fat infiltration according to the 5% cut-off value. A circular region of interest (ROI) of 15 mm was colocalized with the MRS voxel on the corresponding PDFF map.

MRS

Single-voxel MRS (HISTO) with stimulated acquisition mode (STEAM) was performed as the reference. Since T2 decay differs between water and fat, we used high-speed T2 correction to avoid over-evaluation [14]. Five STEAM spectra were produced during a single breath-hold of 15 seconds. A flip angle of 90°C and an ROI of 15 x 15 x 15 mm were used. MRS PDFF values were calculated by the ratio of areas under fat peaks to the sum of the areas underwater and fat peaks.

Elastography

Real-time (supersonic)-two dimensional (2D) shear wave elastography (rt-SWE) was applied as intercostal. Patients were placed in the supine or lateral position with the right arm elevated above the head. Patients were instructed to hold their breath for 5 to 10 seconds while the measurements were made. Colored charts showing liver stiffness were used. In addition to visual assessment of these colored charts, quantitative ROI-based SWE measurement was made in the “stiffest” appearing areas in kilopascals (kPa). The measurements were performed in five different areas of the liver parenchyma and all SWE values were recorded [15].

Ethics statement

Before the beginning of the study, the necessary approval was obtained from the Istinye University Clinical Research Ethics Committee (approval 212019.K-19). All patients who accepted to participate were informed in detail about the objectives of the study and gave written and verbal consent. The study was conducted in accordance with the ethical principles of the Declaration of Helsinki.

Statistical analysis

Data obtained in the study were analyzed using the IBM Statistical Package for Social Sciences (SPSS), version 22.0 software (IBM SPSS Statistics, Armonk, NY). PDFF values of the patients were expressed as mean ± standard deviation. The normality of the data was tested with the Kolmogorov-Smirnov method. Since the data showed no normal distribution, the correlation between MRS and MRI values was examined with Spearman’s correlation analysis. In order to determine the diagnostic accuracy of the MRI-PDFF (based on the MRS-PDFF as a reference), the sensitivity, specificity, positive predictive value, and negative predictive value of the MRI were calculated. The optimum cut-off value was determined using receiver operating characteristic (ROC) analysis. A p-value < 0.05 was considered statistically significant. Spearman's rank correlation coefficient was considered to indicate a weak correlation between 0.2 - 0.4, a moderate correlation between 0.4 - 0.6, a good correlation between 0.6 - 0.8, and an excellent correlation at a value > 0.8.

## Results

A total of 60 patients (30 females (50%), 30 males (50%)) who were referred to our clinic for liver investigations between December 2019 and February 2020 were included in the study. The mean age of the patients was 47.25 ± 14.30 years (range: 24 - 75). The mean age was found as 46.53 ± 13.98 (range: 24 - 72) years in male and 47.97 ± 14.29 (range: 25 - 75) years in female patients. No significant difference was found between the male and female patients (p > 0.05). The mean body mass index (BMI) value of the participants was found as 27.55 ± 5.59 kg/m^2^. Evaluating the patients by BMI values; 20 (33.33%) patients were normal, 25 (45.67%) patients were overweight, and 15 (25%) were obese.

**Figure 1:**
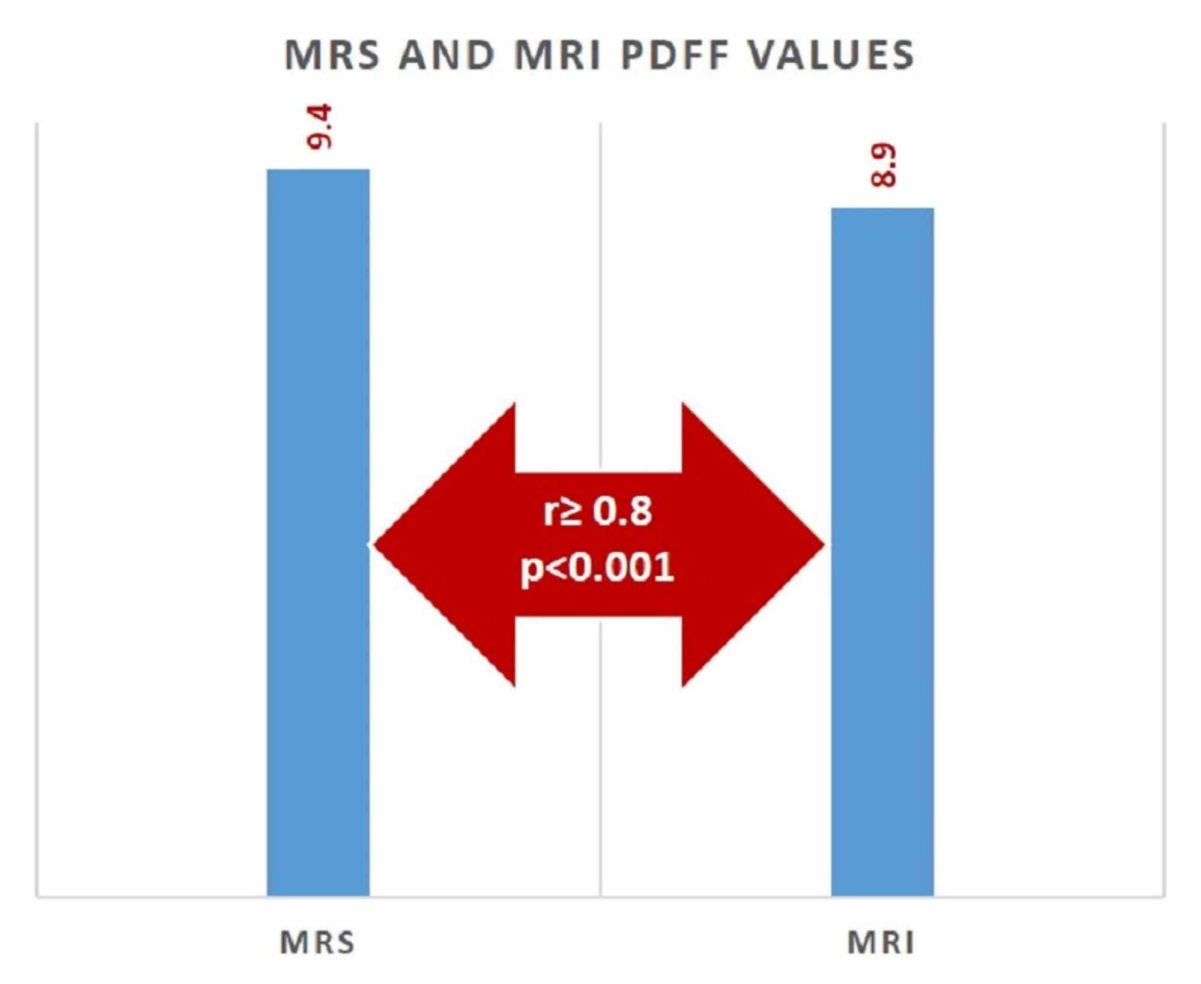
Correlation between MRS and MRI PDFF values for predicting hepatic steatosis MRI: magnetic resonance imaging; MRS: magnetic resonance spectroscopy; PDFF: proton density fat fraction

**Figure 2:**
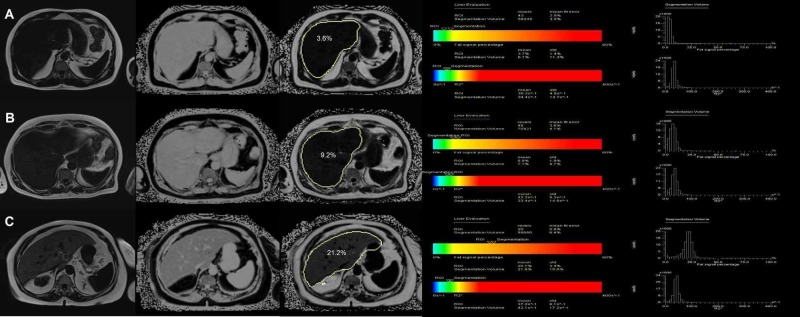
Examples of the multi-echo Dixon (left) and magnetic resonance spectroscopy (right) in three different participants with different percentages of proton density fat fraction values at ROI A) PDFF of 3.6% measured by MRI and 3.7% by MRS in Patient 1; B) PDFF was measured as 9.2% by MRI and 8.8% by MRS in Patient 2; C) PDFF value of 21.2% by MRI and 20.1% by MRS in Patient 3 MRI: magnetic resonance imaging; MRS: magnetic resonance spectroscopy; PDFF: proton density fat fraction; ROI: region of interest

The mean MRI-PDFF value with MRS ROI was found as 9.4% ± 12.1%. The mean MRS-PDFF was found as 8.9% ± 11.3%. No statistically significant difference was found between MRS-PDFF and MRI-PDFF values measured in ROI (p < 0.005) (Figure 1). Examples of the multi-echo Dixon and magnetic resonance spectroscopy with different percentages of PDDF values are given in Figure 2.

The correlation between MRS-PDFF and MRI-PDFF was examined with Spearman’s correlation analysis. Accordingly, there was an excellent correlation between MRS and MRI values measured in ROI (r ≥ 0.8, p < 0.001).

When MRS-PDFF was taken as a reference, sensitivity, specificity, positive predictive value, and negative predictive value were calculated as 96%, 100%, 89.5%, and 92.6%, respectively, for MRI-PDFF in predicting hepatic steatosis for the same ROI localization with MRS. According to the ROC analysis, the optimum cut-off value of MRS-PDFF in predicting hepatic steatosis was found as 5.3% using the same ROI localization with MRS. The area under the curve (AUC) value of MRI-PDFF was found as 0.989 (95% confidence interval (CI): 0.972 - 1.00).

In our study, all patients underwent elastography at the same time in order to determine hepatic steatosis through hepatic tissue stiffness. Stiffness degrees were correlated with MRI-PDFF and MRS PDFF values, although these correlations were not statistically significant (both p > 0.05).

## Discussion

Non-alcoholic fatty liver disease has reached epidemic sizes in most developed countries. The strong association between hepatic fat, metabolic syndrome, and cardiovascular diseases make hepatic steatosis even more important [16].

Hepatic steatosis is typically encountered in radiology practice mostly as an incidental finding or a part of investigations performed for high liver enzymes. Hepatic steatosis can be detected with ultrasonography, but this method is largely subjective and does not provide quantification. In recent years, newer methods have been introduced for this purpose. MRS is accepted as the most accurate method for PDFF measurement. However, its availability is limited, it is not completely supported by the system software of clinical MRI scanners, and MRS requires technical expertise [12]. Although various MR techniques can detect hepatic steatosis, newer PDFF techniques have replaced spectroscopy as a non-invasive reference standard [17]. Comparing with MRS, multi-echo MRI techniques have several advantages in the detection of hepatic steatosis. MRI is more easily available. In addition, MRI can quantify hepatic fat content both in ROI and the entire liver. Therefore, when liver fat is distributed in a nonhomogeneous way, sampling errors are avoided. Furthermore, the image can not be obtained with MRS during breath-holding, and the operator should perform complex processes using specific software [18].

In the present study, the accuracy of the MRI Dixon sequence in predicting hepatic steatosis was investigated based on MRS (HISTO) sequences as a reference. According to the results of this study, we concluded that the use of MRI in the determination of hepatic steatosis is appropriate.

In our study, using the same ROI with MRS, the mean MRI-PDFF value was found as 9.4% and the mean MRS-PDFF value at 8.9%. In a study by Zhao et al. investigating the accuracy of multi-echo Dixon sequence in the detection of hepatic steatosis in Chinese children and adolescents, using the same ROI with MRS, the mean MRI-PDFF was found as 9.9% and MRS-PDFF as 9.1% [19].

In our study, the correlation between MRI-PDFF and MRS-PDFF was examined using Spearman’s correlation analysis. Accordingly, an excellent correlation was found between both methods (r ≥ 0.8, p < 0.001). In a study by Idilman et al., an excellent correlation was observed between these two methods in detecting hepatic steatosis (r = 0.986, p < 0.001) [20]. The authors reported that MRI-PDFF and MRS correctly distinguished moderate/severe steatosis from mild steatosis these two methods were not superior over each other. Again, in a study by Kang et al., a significant correlation was found between the modified Dixon MR technique and MRS [21]. Similar results were reported by Tang et al. and Kukuk et al. [22-23]. Recently, two studies (with one being conducted in adults and the other in children) evaluated patients with known or suspected non-alcoholic fatty liver [24-25]. In these studies, when MRI and MRS measurements were carefully colocalized, the longitudinal change in MRI-PDFF values was found to be closely correlated with longitudinal change in MRS-PDFF values (r = 0.96 and r = 0.986, respectively).

In our study, the sensitivity of MRI in predicting hepatic steatosis was found as 96% and specificity as 100%. The sensitivity and specificity of MRI in detecting mild hepatic steatosis have been reported between 76.7% - 90.0% and 87.1% - 91%, respectively, in the literature [26-27]. In a study by Mazhar et al. who were investigating the non-invasive evaluation of hepatic steatosis, sensitivity and specificity of MRI in detecting mild hepatic steatosis was reported as 85% and 100% [28].

In the present study, the optimum cut-off value of MRI in detecting hepatic steatosis was found to be 5.3%. Zhao et al. reported optimum MRI-PDFF threshold values as 5.1% [19]. However, there are studies reporting lower cut-off values in the literature [10, 29-30]. We attributed the differences in MRI-PDFF cut-off values among the studies to the differences between patient groups included and not including biopsy outcomes in some studies, as in our study.

The strengths of our study are its prospective design, a relatively high number of patients, and evaluation of MRS and MRI findings with elastography at the same time.

The most important limitation of our study was not including liver biopsy outcomes. We think that biopsy in all patients with mild or absent hepatic steatosis may not be reasonable. Finally, we could not compare MRS and MRI findings with biomedical parameters.

## Conclusions

The results of this study indicated an excellent correlation between MRI-PDFF and MRS-PDFF. The multi-echo Dixon MRI technique seems a promising alternative method in the detection of non-alcoholic fatty liver disease. However, the introduction of this relatively newer method into routine practice requires further multicenter and comprehensive studies, including various patient groups.
